# Accuracy of online symptom assessment applications, large language models, and laypeople for self–triage decisions

**DOI:** 10.1038/s41746-025-01566-6

**Published:** 2025-03-25

**Authors:** Marvin Kopka, Niklas von Kalckreuth, Markus A. Feufel

**Affiliations:** Division of Ergonomics, Department of Psychology and Ergonomics (IPA), Technische Universität Berlin, Berlin, Germany

**Keywords:** Health policy, Health services, Public health

## Abstract

Symptom-Assessment Application (SAAs, e.g., NHS 111 online) that assist laypeople in deciding if and where to seek care (*self-triage*) are gaining popularity and Large Language Models (LLMs) are increasingly used too. However, there is no evidence synthesis on the accuracy of LLMs, and no review has contextualized the accuracy of SAAs and LLMs. This systematic review evaluates the self-triage accuracy of both SAAs and LLMs and compares them to the accuracy of laypeople. A total of 1549 studies were screened and 19 included. The self-triage accuracy of SAAs was moderate but highly variable (11.5–90.0%), while the accuracy of LLMs (57.8–76.0%) and laypeople (47.3–62.4%) was moderate with low variability. Based on the available evidence, the use of SAAs or LLMs should neither be universally recommended nor discouraged; rather, we suggest that their utility should be assessed based on the specific use case and user group under consideration.

## Introduction

Symptom-assessment Applications (SAAs, also known as online symptom checkers or digital triage tools) are digital platforms accessible via smartphones or websites that analyze symptoms using various methods^1,2^. For example, some use rule-based algorithms, whereas others use neural networks or simply refer to their algorithm as “Artificial Intelligence” (AI) without further specification^3,4^. They provide a diagnosis and recommendation regarding whether and where medical care should be sought, a process known as *self-triage*^2^. Self-triage recommendations differ from diagnostic recommendations in their focus. Whereas diagnostic recommendations aim to assign a diagnosis to symptoms and do not seem to be useful for medical laypeople, a self-triage recommendation on the next course of action can help patients in determining the urgency of their symptoms and finding an appropriate healthcare facility^2,5^. For example, a person using an SAA who experiences severe chest pain and breathlessness would be advised to seek emergency care, even if the underlying condition remains undiagnosed at the point of app use^2^. SAAs are potentially useful for various stakeholders: health protection agencies may use the symptom input for syndromic surveillance^6^, general practitioners and clinics can implement SAAs for patient (re-)direction^7,8^, and medical laypeople can use them for assistance in health-related care-seeking decisions^9^. Hence, they could improve the efficient distribution of healthcare resources and ultimately increase healthcare access and health equity by providing health advice and recommendations regardless of a person’s socioeconomic status, education, or other social determinants of health.

SAAs are increasingly used worldwide. For instance, the United Kingdom’s National Health System (NHS) launched *NHS 111 online* in 2017^10^ and Germany’s Association of Statutory Health Insurance Physicians supplemented their triage hotline with the digital *PatientenNavi* in 2021^11,12^. Consequently, these tools perform millions of assessments annually, with about 7% of the German population using SAAs^10,13^. However, some studies raised concerns about their real-world utility and cost-effectiveness, as they did not seem to reduce healthcare utilization in an NHS evaluation study^14^. This is no surprise, as SAAs tend to be risk-averse and frequently provide users with a recommendation of higher urgency than necessary, making them seek care more often than necessary (over-triage)^2,15^. As a logical result of this tendency to *over-triage*, fewer users are *under-triaged*, that is, fewer users receive a recommendation of lower urgency than warranted, reducing potential safety risks to users^15,16^. Hence, both the safety and accuracy of SAAs have been subjects of several studies. Three systematic reviews have been published to synthesize the available evidence on SAAs so far and show that, although most studies had a high risk of bias, SAA accuracy generally tends to be far from perfect, and performance varies greatly between different apps^17–19^.

As an alternative to SAAs, Large Language Models (LLMs) have been proposed^16,20^. After becoming available in 2022, they quickly garnered interest in the medical community because they were good enough to pass state licensing exams and, as a result, are now suggested as potential clinical decision support systems^21–23^. Some studies have also tested LLMs with cases developed for SAAs and suggest them as decision support tools for medical laypeople^24,25^. Nevertheless, an evidence synthesis that reports the accuracy of LLMs for self-triage decisions is still missing.

All these studies on SAAs and LLMs have in common that they view these tools as the sole decision-makers, and, based on this research, researchers may recommend or discourage their use without considering the accuracy of actual users as baseline. This perspective might overlook scenarios where users alone perform poorly and even suboptimal SAAs could be beneficial. Conversely, if users generally make good decisions, SAAs might not offer any effective assistance. Although one study compared the accuracy of SAAs directly with that of laypeople^26^, an evidence synthesis contextualizing the accuracy of SAAs and LLMs with the accuracy of laypeople is missing.

Therefore, this systematic review aims to extend previous reviews on SAA accuracy^17–19^ by including studies on the accuracy of LLMs as an alternative to SAAs and the baseline accuracy of medical laypeople as the intended users of these tools. This comparison shifts the focus from SAAs and LLMs as the sole decision-making entity to considering the user group of medical laypeople as a benchmark against which their accuracy should be interpreted. Because specific diagnoses are of little to no use for medical laypeople, this review focuses on the most relevant use case of SAAs and LLMs—self-triage decisions—and deliberately excludes diagnostic accuracy.

## Results

### Included studies

**Fig. 1:**
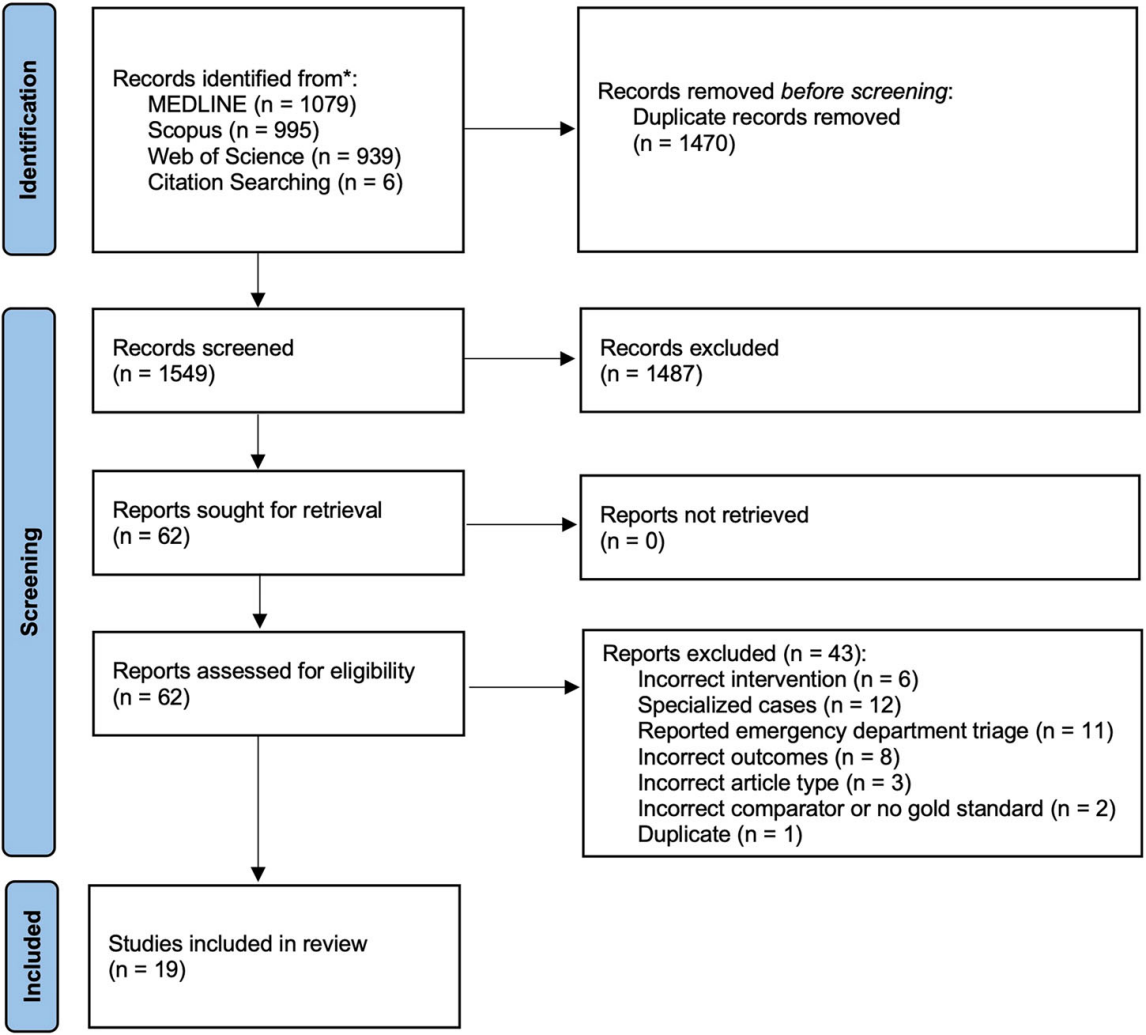
PRISMA flow diagram detailing the study search and selection process. Overall, 1470 studies were screened and 19 studies included.

In total, 3019 potentially eligible studies were identified (3013 using the database search and 6 using citation search). After excluding ineligible studies, for example because they referred to emergency department triage only^27^, 19 studies were included in the review, see Fig. 1.

**Fig. 2:**
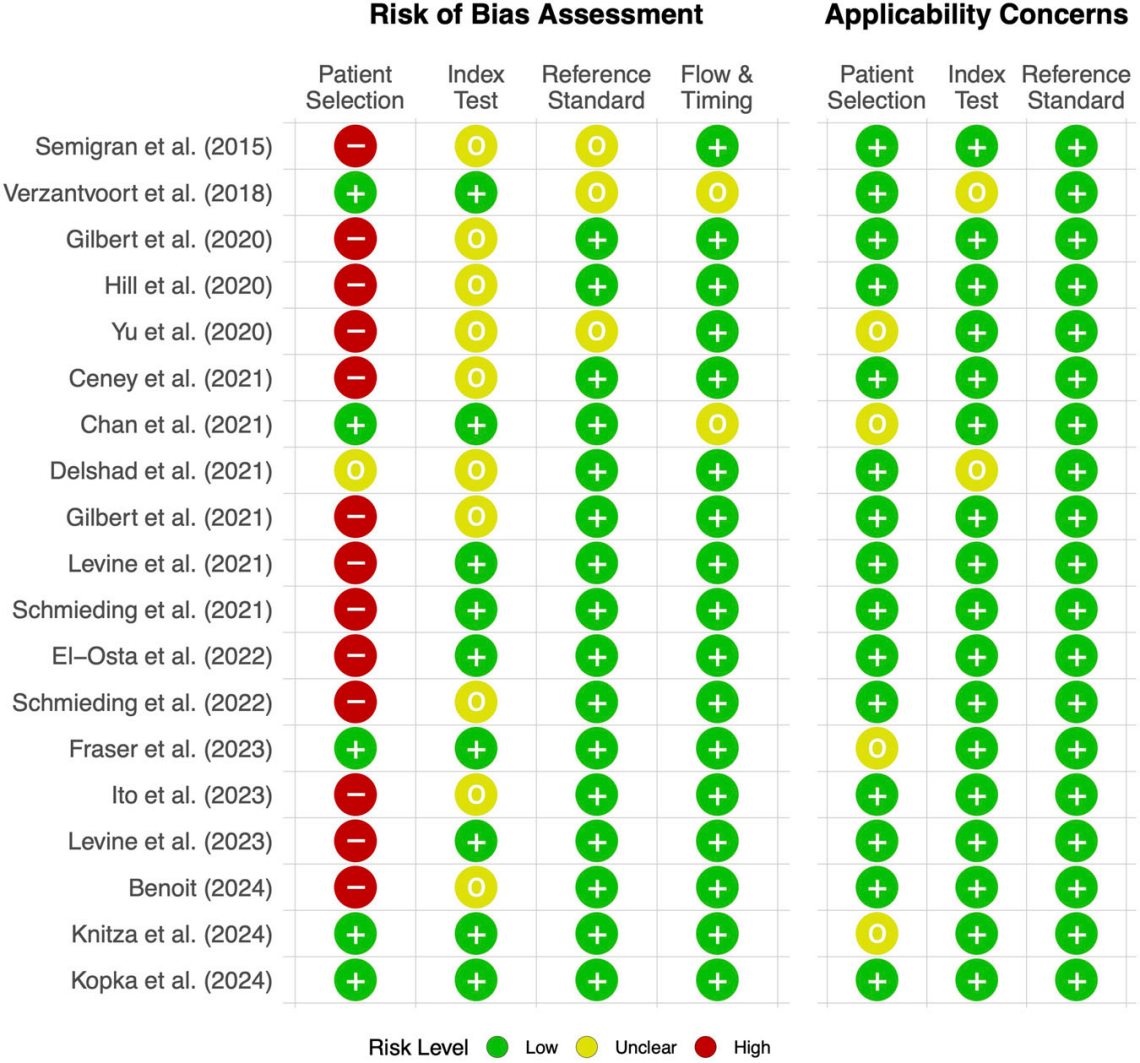
Risk of bias assessment and applicability concerns using QUADAS-2. Patient selection generally had the highest risk of bias among all studies. Index test mostly had low or unclear risk of bias. Reference standard mostly had low risk of bias with 3 studies having unclear risk of bias. Flow & timing only had 2 studies with unclear risk of bias. Applicability concerns were generally low.

Most included studies (89%, 17/19) had at least one area with a high or unclear risk of bias, see Fig. 2. The area with the highest risk of bias was patient selection as most studies used fictitious but clear-cut vignettes to assess triage accuracy. For example, Semigran et al. used cases from textbooks and other medical resources with a given diagnosis^2^, and other studies used cases that were based on clinicians’ experience^28–30^. Both methods yield cases that are not representative of the more ambiguous and less clear-cut cases that people tend to enter into SAAs^20,31,32^. Only five studies had a low risk of bias because they included real patient cases: three studies used cases based on patients seeking care in emergency departments and primary care settings^16,33,34^, one study directly surveyed SAA users^7^, and one study derived patient cases from medical laypeople who were making self-triage decisions and sought assistance for their decision online^20^.

Index test was another area in which many studies (53%, 10/19) have some risk of bias. Most of them did not report blinding of the inputter^2,4,15,24,25,29,35–38^. One study did not report how results from SAAs were obtained at all^35^. With respect to the reference standard used for performance evaluation, only few studies have a moderate or high risk of bias (16%, 3/19). Those with concerns did not report how their gold standard was determined or used the judgment of one person only, e.g., the triage nurse in the emergency department^2,7,36^. Studies with unclear risk regarding flow and timing of the assessment procedure had follow-up contact several hours after a patient had used an app^7^ or did not mention when cases were reviewed^34^.

Applicability concerns were generally low. Most concerns comprised case or vignette selection in studies that only used cases from the emergency department or a general practitioner setting, without including self-care cases^16,33,34,36^. Two studies had unclear applicability of the index test, as one study used binary decisions only (visit a medical professional or not)^7^, and another study did not provide information how SAA results were determined^35^.

### Study characteristics

In total, 14 (74%) studies analyzed the self-triage accuracy of SAAs^2,4,7,15,16,20,29,33–39^, four (21%) of the studies focused on the accuracy of laypeople^20,26,30,34^, and four (21%) studies on the accuracy of LLMs^20,24,25,28^. For SAAs, three (21%) studies let patients enter their symptoms directly^7,33,34^, three (21%) used real patient cases that were entered retrospectively^16,20,36^, and the remaining 8 (57%) studies used fictitious case vignettes developed by medical professionals^2,4,15,29,35,37–39^. For studies on laypeople, one study (25%) asked participants how they would rate the urgency of their own symptoms^34^, one (25%) used real patient cases that were presented to laypeople^20^ and two (50%) used fictitious vignettes phrased by medical professionals^26,30^. For LLMs, no study let patients enter symptoms themselves, one (25%) used real patient cases retrospectively^20^ and three (75%) used fictitious vignettes^24,25,28^.

Six (43%) studies examined only one SAA^7,38,39^, two (14%) studies examined two SAAs^16,36^ and six (43%) studies examined multiple SAAs^2,4,15,20,29,37^, ranging from seven^29^ to 23 different SAAs^2^. Studies on laypeople used sample sizes between 91 participants^26^ and 5,000 participants^30^. For LLMs, three (60%) studies examined only one LLM^24,25,28^, whereas two (40%) studies examined multiple LLMs, ranging from two^16^ to five models^20^. The most frequently included SAA was Ada Health and the most frequently included LLM was GPT-4. Six out of 14 (43%) studies reported at least some information on how the evaluated SAAs functioned^2,4,7,34,35,37^. Five of these (83%) included SAAs that were built on rule-based algorithms^2,4,7,34,37^, whereas three (50%) included SAAs that claimed to use AI^4,35,37^. The general training data or data basis of the tools was mentioned in four (67%) studies^4,7,34,35^ and comprised either established triage guidelines like the Thompson guideline^2^ or the Dutch Triage System^7^ or was built on a self-developed medical knowledge base^16,34,35^. However, no study gave specific in-depth details that would allow researchers to examine the training data. All study characteristics are summarized in Table 1. A PICOS overview can be found in Table 2.

**Table 1:** Characteristics of the included studies

Authors (year)	Study Design	Description of cases	Number of SAAs, laypeople, and LLMs	Number of cases or vignettes
Semigran et al.^2^.	Cross-sectional vignette study	Fictitious cases derived from various medical resources	SAAs: 23, Laypeople: None, LLMs: None	45
Verzantvoort et al.^7^.	Prospective cross-sectional cohort study	Patients used an app and entered their own symptoms	SAAs: 1, Laypeople: None, LLMs: None	126
Gilbert et al. ^29^.	Cross-sectional vignette study	Vignettes were created based on NHS triage calls (32%) and supplemented with fictitious vignettes developed from medical professionals (68%)	SAAs: 7, Laypeople: None, LLMs: None	200
Hill et al.^4^.	Cross-sectional vignette study	Fictitious cases from Semigran et al. extended to include Australia-specific vignettes	SAAs: 19, Laypeople: None, LLMs: None	48
Yu et al.^36^.	Retrospective cohort study	Real cases from the emergency department were transcribed to case vignettes	SAAs: 2, Laypeople: None, LLMs: None	100
Ceney et al. ^37^.	Cross-sectional vignette study	Fictitious case vignettes from Semigran et al.	SAAs: 10, Laypeople: None, LLMs: None	50
Chan et al.^34^.	Prospective cohort study	Patients in the emergency department and family practices entered their symptoms into an app	SAAs: 23, Laypeople: 581, LLMs: None	581
Delshad et al. ^35^.	Cross-sectional vignette study	Fictitious case vignettes were developed	SAAs: 1, Laypeople: None, LLMs: None	50
Gilbert et al. ^38^.	Cross-sectional vignette study	Fictitious case vignettes from Hill et al.	SAAs: 1, Laypeople: None, LLMs: None	48
Levine et al. ^30^.	Cohort study	Fictitious case vignettes developed based on Semigran et al. and Hill et al.	SAAs: None, Laypeople: 5000, LLMs: None	48
Schmieding et al.^26^.	Longitudinal vignette study	Fictitious case vignettes from Semigran et al.	SAAs: None, Laypeople: 91, LLMs: None	45
El-Osta et al. ^39^.	Cross-sectional vignette study	Fictitious vignettes created by medical professionals	SAAs: 1, Laypeople: None, LLMs: None	139
Schmieding et al.^15^.	Cross-sectional vignette study	Fictitious case vignettes from Semigran et al.	SAAs: 17, Laypeople: None, LLMs: None	45
Fraser et al. ^16^.	Clinical data analysis	Patients in an emergency department entered their symptoms. Reports from the app were used to evaluate the tools	SAAs: 2, Laypeople: None, LLMs: 2	37
Ito et al.^25^.	Cross-sectional vignette study	Fictitious case vignettes from Semigran et al.	SAAs: None, Laypeople: None, LLMs: 1	45
Levine et al. ^28^.	Cross-sectional vignette study	Fictitious case vignettes developed based on Semigran et al. and Hill et al.	SAAs: None, Laypeople: None, LLMs: 1	48
Benoit (2024)	Cross-sectional vignette study	Fictitious case vignettes from Semigran et al.	SAAs: None, Laypeople: None, LLMs: 1	45
Knitza et al. ^33^.	Cross-over randomized trial	Patients in the emergency department entered their symptoms	SAAs: 1, Laypeople: None, LLMs: None	437
Kopka et al. ^20^.	Retrospective cohort study	Real patient cases from an ‘ask the doctor’ platform where laypeople asked for help in their self-triage decision	SAAs: 12, Laypeople: 198, LLMs: 5	45

**Table 2:** PICOS information of the included studies

Authors (year)	Population	Intervention	Comparator	Outcomes	Study Design
Semigran et al.^2^.	General patient population	23 SAAs	No information	Accuracy	Cross-sectional vignette study
Verzantvoort et al.^7^.	Primary care patients	1 SAA	Triage nurse	Accuracy, Sensitivity, Specificity, Positive Predictive Value, Negative Predictive Value, Confusion Matrix	Prospective cross-sectional cohort study
Gilbert et al. ^20^.	NHS triage callers and general patient population	7 SAA	Physician panel	Accuracy, Comprehensiveness, Safety, Confusion Matrix	Cross-sectional vignette study
Hill et al.^4^.	General patient population	19 SAAs	Two physicians and one emergency specialist	Accuracy	Cross-sectional vignette study
Yu et al.^36^.	Emergency department patients	2 SAAs	Triage nurse	Accuracy, Over-/Undertriage, Sensitivity, Specificity, Positive Predictive Value, Negative Predictive Value, Confusion Matrix	Retrospective cohort study
Ceney et al. ^37^.	General patient population	10 SAAs	Comparator taken from Semigran et al.	Accuracy, Comprehensiveness, Safety	Cross-sectional vignette study
Chan et al. ^34^.	Emergency department and family physician cases	23 SAAs, 581 laypeople	Treating physician, decisions reviewed by two external physicians	Accuracy, Over-/Undertriage, Confusion Matrix	Prospective cohort study
Delshad et al. ^35^.	General patient population	1 SAA	Consensus from several physicians	Accuracy	Cross-sectional vignette study
Gilbert et al. ^38^.	General patient population	1 SAA	Comparator taken from Hill et al.	Accuracy	Cross-sectional vignette study
Levine et al. ^30^.	General patient population	5000 laypeople	Two physicians	Accuracy	Cohort study
Schmieding et al.^26^.	General patient population	91 laypeople	Comparator taken from Semigran et al.	Accuracy, Over-/Undertriage	Longitudinal vignette study
El-Osta et al. ^39^.	General patient population	1 SAA	General practitioners (GPs) that developed vignettes and three independent GPs	Accuracy, Safety	Cross-sectional vignette study
Schmieding et al.^15^.	General patient population	17 SAAs	Comparator taken from Semigran et al.	Accuracy, Over-/Undertriage, Binary Self-Triage Decision, Confusion Matrix	Cross-sectional vignette study
Fraser et al. ^16^.	Emergency department patients	2 SAAs, 2 LLMs	Three emergency physicians	Accuracy, Safety, Overcaution	Clinical data analysis
Ito et al.^25^.	General patient population	1 LLM	Comparator taken from Semigran et al.	Accuracy	Cross-sectional vignette study
Levine et al. ^28^.	General patient population	1 LLM	Two physicians	Accuracy	Cross-sectional vignette study
Benoit (2024)	General patient population	1 LLM	Comparator taken from Semigran et al.	Accuracy	Cross-sectional vignette study
Knitza et al. ^33^.	Emergency department patients	1 SAA	Two physicians	Accuracy, Over-/Undertriage, Confusion Matrix	Cross-over randomized trial
Kopka et al. ^20^.	Medical laypeople seeking online decision support for general symptoms	12 SAAs, 198 laypeople, 5 LLMs	Two physicians	Accuracy, Safety, Over-/Undertriage, Comprehensiveness, Capability Comparison Score	Retrospective cohort study

### Self-triage accuracy

**Fig. 3:**
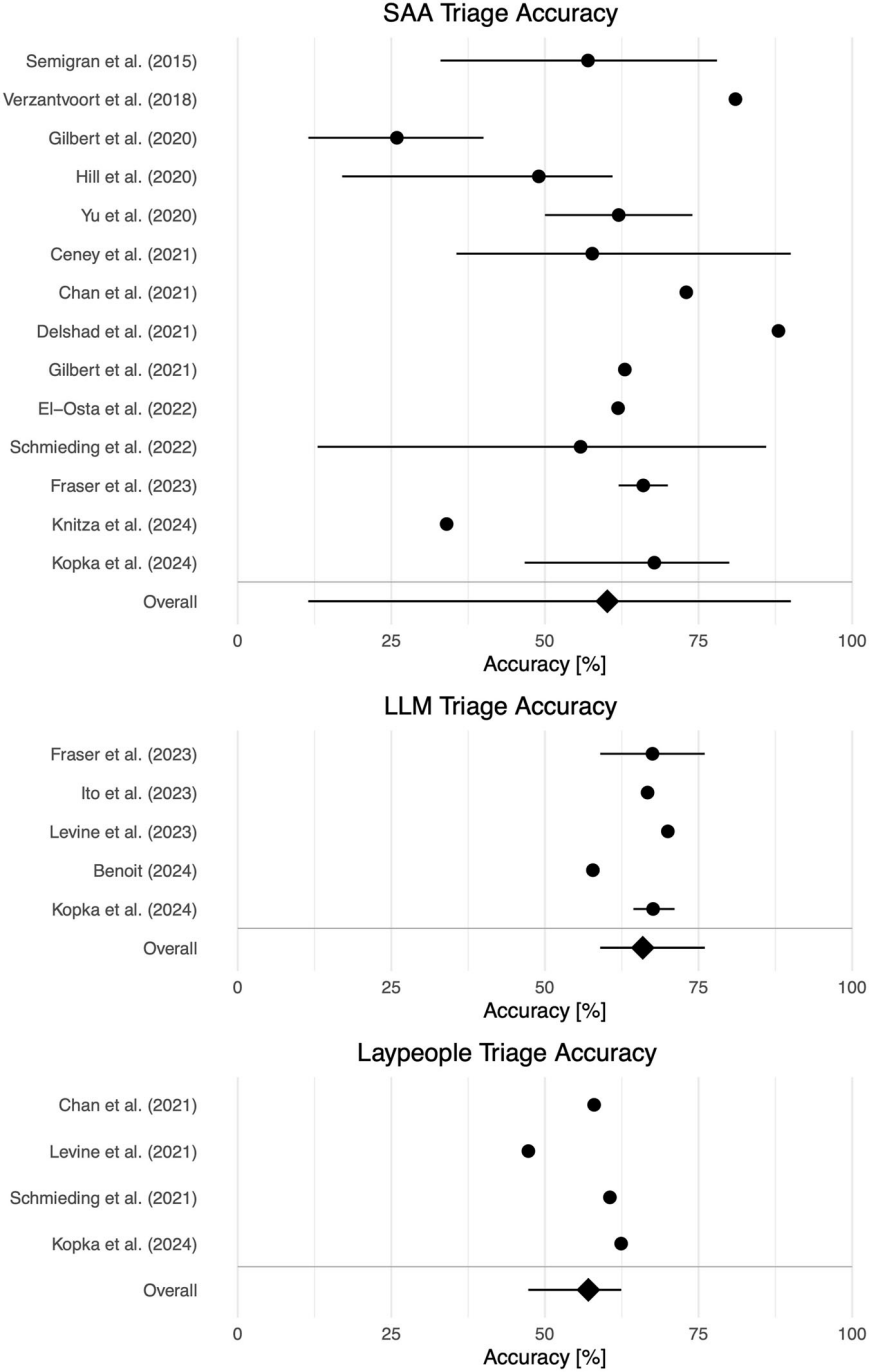
Overview of reported self-triage accuracy estimates for Symptom-Assessment Applications (SAAs), laypeople and Large Language Models (LLMs). Points indicate the reported mean, and lines indicate reported minimum and maximum accuracy values within a study. Studies on laypeople reported means without information on minimum and maximum values. SAA triage accuracy had a high range from 26% to 88%. LLM triage accuracy was the highest with a low range from 58% to 70%. Laypeople’s triage accuracy was the lowest and had a low range from 47% to 62%.

The reported average accuracy of SAAs ranged from 25.9% in a study by Gilbert et al.^29^ to 88.0% in a study by Delshad et al.^35^, see Fig. 3. However, the self-triage accuracy varies widely between different systems: The lowest individual SAA accuracy of 11.5% was reported in the study by Gilbert et al.^29^, whereas the highest accuracy of 90.0% was reported in a study by Ceney et al.^37^.

The average accuracy of LLMs ranged from 57.8% in a study by Benoit^24^ to 70.0% in a study by Levine et al.^28^. Individual accuracy estimates for LLMs had a relatively low variation compared to SAAs and ranged from 57.8% in the study by Benoit^24^ to 76.0% in a study by Fraser et al.^16^.

The reported average accuracy of laypeople also had a lower variation and ranged from 47.3% to 62.4%, see Fig. 3. No study reported the accuracy of individual laypeople, making a comparison of worst- and best-performing individuals with SAAs and LLMs impossible.

Most studies also reported average accuracy across different self-triage levels. For all three agents, accuracy differed between these urgency levels. SAAs generally had a high accuracy for emergency cases (74.5%, with a range from 57% to 100%) and a lower accuracy for urgent cases (53.3% range from 23.0% to 92.2%) and non-emergent cases (69.7%, range from 55.0 to 82.5%)^2,33,34,36^. Their accuracy was the lowest for self-care cases (42.1%, range from 0.0% to 74.0%)^7,33^.

LLMs had a moderate to high accuracy in emergency cases (66.7%, range from 50% to 86.7%) and reliably identified non-emergency cases (94.1%, range from 87% to 100%)^20,24,25,28^. However, they had a very low accuracy for self-care cases (10.8%, range from 6.15% to 16.7%)^20,28^.

Laypeople had a relatively high accuracy in identifying emergency cases (67.9%, range from 57.5% to 78.6%) and non-emergency cases (70.8%, range from 68.4% to 73.2%)^20,26,30^. For self-care cases, they had a low accuracy (35.6%, range from 25.4% to 46.7%)^26,30^, see Table 3.

**Table 3:** Reported self-triage accuracy of Symptom-Assessment Applications, Laypeople, and Large Language Models across different self-triage levels

Self-Triage Level	Symptom-Assessment Applications, % (Range)	Large Language Models, % (Range)	Laypeople, % (Range)
Emergency	74.5% (57–100%)	66.7% (50.0–86.7%)	67.9% (57.5–78.6%)
Urgent Care	53.3% (23.0–92.2%)	16.7% (n.a.)	50.0% (n.a.)
Non-Emergency/ Non-Urgent	69.7% (55.0–82.5%)	94.1% (87–100%)	70.8% (68.4–73.2%)
Self-Care	42.1% (0.0–74.0%)	10.8% (6.15–16.7%)	35.6% (25.4–46.7%)

**Fig. 4:**
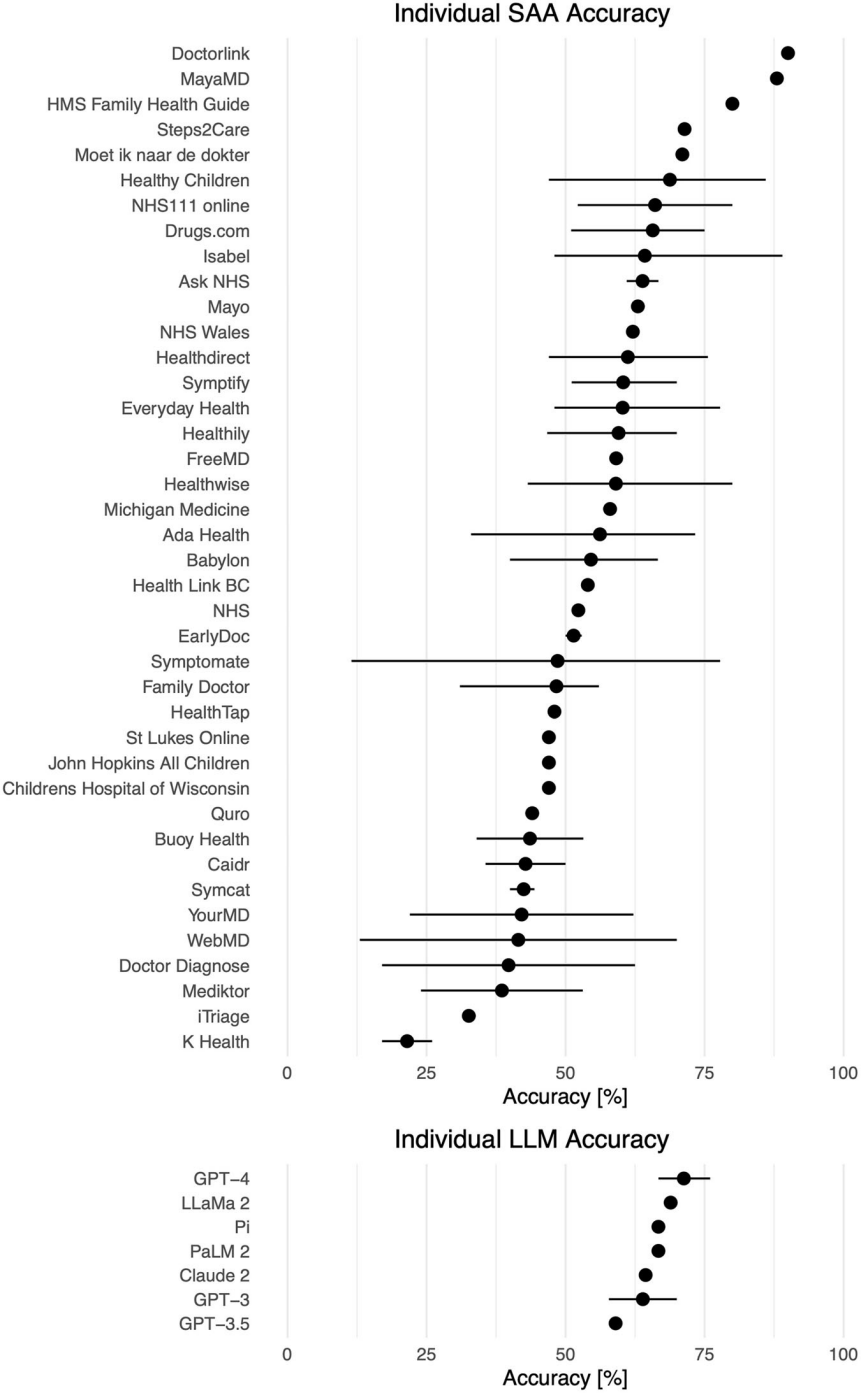
Overview of accuracy values reported for individual Symptom-Assessment Applications (SAAs) and large language models (LLMs) across multiple studies. Points indicate the mean of reported accuracy values, and lines indicate minimum and maximum reported accuracy values. SAAs and LLMs without a line were examined in one study only. Since the methodology between studies differ and some are sponsored by the developer, the accuracy of these SAAs/LLMs should be interpreted with caution. DoctorLink showed the highest accuracy, but was tested in one study only, whereas K Health had the lowest accuracy in multiple studies. GPT-4 showed the highest accuracy among LLMs and GPT-3.5 had the lowest accuracy.

Individual SAAs demonstrated highly variable accuracies: Doctorlink–which was examined in one study only–had the highest accuracy with 90.0%, whereas K Health had the lowest accuracy with 21.5%. When only examining SAAs that were tested across multiple studies, Healthy Children (68.8%, 47.0–73.3%) and NHS111 online (66.1%, 52.2–80.0%) had the highest accuracy among all SAAs. The spread between the accuracy reported in different studies for the same SAA was high as well. For example, accuracy values for Symptomate ranged from 11.5% to 77.8% (with a mean of 48.6%), see Fig. 4.

For LLMs, the spread was relatively low. Although GPT-4 had the highest accuracy (71.3%), all LLMs scored between 59.0% and 71.3%. The accuracy between different studies only ranged from 66.7% to 76.0% for GPT-4, and 57.8% to 70.0% for GPT-3.

### Methodology

The methodology varied between studies. Most studies assigned the gold standard for each case using a physician panel of two or more physicians who independently rated the cases and resolved disagreements through discussion^4,20,33^. Other studies omitted independent ratings and directly used a physician discussion panel without letting them rate cases independently beforehand^29,35^. Alternatively, in some studies, the authors (who are physicians) assigned the gold standard themselves^28,30^ or used the decision of a single triage nurse^7,36^. Most studies used only one person to input data into SAAs and LLMs^2,4,15,16,24,25,28,37,38^. Two studies employed two people^20,36^, one study six people^39^ and one study eight people^29^. Some studies used medical professionals as inputters^2,29^, whereas others asked laypeople to enter the symptoms^15,20^. Notably, only two studies mentioned blinding inputters to the gold standard solution^16,20^. Although most studies used three self-triage levels in their assignment^2,15,16,20,24–26^, some used only two^7,36^ (e.g., emergency or no emergency), and one study used six levels^29^.

The methodology also varied in terms of self-triage outcomes that were reported. In addition, to mean accuracy, two studies used metrics from signal detection theory (sensitivity, specificity, positive predictive value and negative predictive value)^7,36^, three studies reported the comprehensiveness of an SAA^20,29,37^, seven studies reported the inclination to over- and undertriage^15,16,20,26,33,34,36^, five studies reported the safety of advice^16,20,29,37,39^, and six studies reported a confusion matrix^7,15,29,33,34,36^. One study additionally reported the Capability Comparison Score, which was developed specifically to compare SAAs that differ with respect to the kinds and difficulties of vignettes it can or cannot address^20,40^, see Table 4.

**Table 4:** Methodological details of the included studies

Authors (year)	Gold Standard	Number of inputters	Number of self-triage levels	Other outcomes reported
Semigran et al.^2^.	Correct diagnosis was part of medical resource; no information on self-triage level	1	3	None
Verzantvoort et al.^7^.	Triage nurse determined self-triage level after telephone interview	n.a.	2	Sensitivity, Specificity, Positive Predictive Value, Negative Predictive Value, Confusion Matrix
Gilbert et al. ^29^.	Assigned by physician panel	8	6	Comprehensiveness, Safety, Confusion Matrix
Hill et al.^4^.	Two physicians and one emergency specialist rated cases, disagreement resolved through discussion	1	4	None
Yu et al.^36^.	Assigned triage level by triage nurse upon visiting emergency department	2	2	Over-/Undertriage, Sensitivity, Specificity, Positive Predictive Value, Negative Predictive Value, Confusion Matrix
Ceney et al. ^37^.	Taken from Semigran et al. and assessed against National Institute for Health and Care Excellence summaries	1	4	Comprehensiveness, Safety
Chan et al. ^34^.	Decision from treating physician was reviewed by two physicians	n.a.	4	Over-/Undertriage, Confusion Matrix
Delshad et al. ^35^.	Several physicians from different institutions were asked about the most appropriate self-triage level. They were asked to develop a consensus	n.a.	4	None
Gilbert et al. ^38^.	Taken from Hill et al.	1	4	None
Levine et al. ^30^.	Assigned by two physicians	n.a.	4	None
Schmieding et al.^26^.	Taken from Semigran et al.	n.a.	3	Over-/Undertriage
El-Osta et al. ^39^.	Multiple gold standards tested: general practitioners (GPs) that developed vignettes also assigned solutions. 3 independent GPs were asked about correct self-triage level. Both solutions were pooled	6	3	Safety
Schmieding et al.^15^.	Taken from Semigran et al.	1	3	Over-/Undertriage, Binary Self-Triage Decision, Confusion Matrix
Fraser et al. ^16^.	Three emergency department physicians rated each case	1	3	Safety, Overcaution
Ito et al.^25^.	Taken from Semigran et al.	1	3	None
Levine et al. ^28^.	Assigned by two physicians	1	4	None
Benoit (2024)	Taken from Semigran et al.	1	3	None
Knitza et al. ^33^.	Two physicians rated each case	n.a.	4	Over-/Undertriage, Confusion Matrix
Kopka et al. ^20^.	Panel of 2 physicians rated independently, disagreement resolved through discussion	2	3	Safety, Over-/Undertriage, Comprehensiveness, Capability Comparison Score

## Discussion

This systematic review aimed to synthesize available evidence on self-triage accuracy of SAAs, LLMs, and laypeople as the user group of these technologies. Our findings indicate that SAAs have a relatively low accuracy on average, but they also show that accuracy is highly dependent on the specific tool used. Most studies report a high spread of accuracies between different SAAs, and there is also high heterogeneity between the studies. However, when assessing individual SAAs across different studies, some tools seem to consistently perform well. For example, NHS 111 online was included in multiple studies and consistently showed moderate to high accuracy. Conversely, Mediktor showed a consistently low performance across multiple studies. Surprisingly, LLM accuracy does not have a high spread in comparison. All studies testing LLMs report accuracy values between 58% and 76%, and the individual spread for LLMs across studies is minimal. The same holds true for laypeople. The included studies report accuracies between 47% and 62%, indicating that laypeople make decisions better than chance level but far from perfect. In the following, we discuss the most important findings of our systematic review.

First, our review, although it includes more recent studies, aligns with the findings from previous systematic reviews. These reviews consistently report that SAA accuracy is relatively low, but note that the variability between the tools is very high^17–19^. This variation is understandable, considering that SAAs are developed by different institutions, each using different technologies and working with varying levels of funding. For example, some developers use simple rule-based algorithms, while others use Bayesian networks or algorithms^3^. Based on varying accuracy levels, all reviews conclude that SAAs pose a safety risk and suggest that their use might not be encouraged. For example, Wallace et al. consider the high variability in accuracy a safety hazard and call for a non-inferiority trial comparing SAA usage to the current way laypeople make self-triage decisions^18^. Similarly, Chambers et al. note the absence of evidence on SAA safety and a lacking representativeness of cases they are tested with^17^. Although the safety concerns are valid and important, it is noteworthy that previous systematic reviews did not include data on laypeople’s self-triage accuracy. Our updated review includes the self-triage performance of laypeople and finds that they tend to make self-triage decisions with only moderate accuracy when unassisted. Hence, laypeople may benefit from triage assistance. In fact, our systematic review provides some evidence that some SAAs may actually help to improve laypeople’s self-triage performance because they performed better than laypeople. Thus, more research on how to improve SAAs’ comparative performance seems warranted.

A second finding is a lack of research into interactions between users and tools and their impact on self-triage accuracy. For instance, no study was included in our systematic search that specifically compared laypeople’s performance in combination with SAAs and other options such as triage hotlines or web searches. Because the underlying human-technology interaction is not fully understood, there is a risk that errors of laypeople and decision support technologies may multiply, leading to even worse decisions than if decisions are made separately. Alternatively, correct decisions might complement each other and increase the overall self-triage accuracy beyond the accuracy of each agent alone. Since humans make the final decision in the end, it is also important to understand how they include and compensate incorrect advice. One previous study on human-SAA interaction suggests that laypeople can increase their accuracy with well-performing SAAs, but not to the level of the SAA’s isolated accuracy^41^. However, users were able to compensate incorrect recommendations and were not entirely dependent on the system. Thus, this study suggests that errors do not add up, but rather that laypeople can successfully use SAAs–even if the system’s accuracy is not perfect–and compensate incorrect recommendations. Despite these promising results, we agree with the previous systematic reviews by Wallace et al. and Chambers et al. that the interaction between laypeople and digital tools and their impact on a combined self-triage accuracy warrants further investigation.

Third, the review suggests that it is not universally advisable to recommend or dismiss using SAAs or LLMs. Rather, recommendations should depend on the specific implementation *use case*. When comparing SAAs, LLMs and laypeople, it is important to examine the specific decisions that are made. The accuracy of all three agents differed drastically between the urgency levels of the presented cases. Whereas all performed relatively well in identifying emergencies (with laypeople and SAAs showing very similar accuracy), their accuracy in self-care cases varied considerably. SAAs had a variation between 0 and 74%, while laypeople solved between 25% and 47% of these cases correctly. LLMs rarely advised self-care and thus had an accuracy below 20%. These findings indicate that laypeople may not require assistance in identifying emergencies but could profit from support in identifying self-care cases. However, LLMs are not well-suited for this task and only certain SAAs can be helpful in this regard. Some previous studies suggest dividing the urgency levels into two steps to better reflect how laypeople make self-triage decisions: First, they determine whether their symptoms require medical attention at all, and if so, they then decide where to seek care^15,42^. Considering our findings, laypeople may need more assistance in determining whether their symptoms require medical attention rather than deciding where to seek care; this seems to be the decision in which SAAs and other tools could be most beneficial. When deciding between emergency and non-emergency care, LLMs might be helpful due to their high accuracy in this regard. However, when deciding if care is needed at all, LLMs generally do not offer any assistance, and only some SAAs are useful. Thus, based on the systematic review, a general recommendation for or against SAAs or LLMs cannot be made. However, some tools might be helpful depending on the specific decision they need to make. For instance, when deciding between emergency and non-emergency care, LLMs and specifically GPT-4 might be beneficial, as it has been found to be relatively safe and accurate in this decision^16,20^. On the other hand, if users want to determine whether their symptoms warrant any medical attention at all, using a tool like NHS 111 online could be helpful due to its high accuracy in this decision. Nevertheless, users should always use these tools with caution and verify the recommendations with additional information sources and critical thinking.

The fourth implication relates to necessary methodological improvements to help move the field forward. For evaluators such as researchers, implementers, or policymakers, a standardized evaluation process is essential. However, a first issue of the identified studies is that they report different performance metrics. Although all studies report mean accuracy, this metric is highly influenced by the number and difficulty of the cases that can be entered. Similarly, if diseases are tested whose prevalence is low in the general population, accuracy estimates of an SAA’s or LLM’s performance may be biased and generalize only to specific populations. Additional metrics reported across the included studies differ as well. Some studies used two triage levels only and could report signal detection theory metrics (sensitivity, specificity, positive predictive value and negative predictive value). Although not included in these studies, there are more metrics for binary decisions that are commonly used: precision, recall, the F1 score, and (area under the curve of) receiver operating characteristics (AUROC/ROC) graphs^43^. These might be used in future studies to make results comparable across different digital health tools.

A second issue limiting comparability between studies is the definition of safety. Some authors define recommendations as safe if they are correct or more urgent than the gold standard^20,39^, whereas others define them as safe if they do not lead to adverse events^17^. Still, other authors call for a more nuanced approach by analyzing the exact reason for unsafe advice (e.g., mistaking myocardial infarction for a panic attack)^18,44^ or assessing the psychological harm that could result from overcautious recommendations (e.g., high-urgency advice may be safe for patients from a systems point of view, but cause unnecessary fears in individual users)^45,46^. Thus, moving forward, the field should develop a definition of safety that encompasses all proposed aspects. For example, a multi-layered safety concept could include treatment safety (advice being safe in terms of causing no harm due to delayed treatments), adverse events, and psychological safety (i.e., no unnecessary distress or fears). As a first step, a systematic review could be undertaken that specifically examines the safety of SAA and its definition in different studies. After defining a multi-layered safety concept, it could be measured using experience sampling or field experimental methods again by including not only SAAs and users but also the healthcare facility users went to so that possible adverse events and safety risks can be determined. We also recommend that future evaluation studies should be based on a standardized set of performance metrics that allow a fair comparison across multiple tools and studies (for an example, see Kopka and colleagues’ RepVig framework)^20^.

In addition to the lack of standardized performance metrics, a primary risk in current evaluation studies is the use of fictitious case vignettes^47^. Although these vignettes are convenient and resource-efficient, they often yield results that are not generalizable to real-world settings^20,47^. A cost-effective alternative could involve using real patients’ descriptions of complaints that are entered into SAAs. A procedure for how this can be done is also provided in the RepVig framework^20^. The next step would be to test SAAs and LLMs with actual patients in a clinical trial to validate positive findings based on vignette-based studies. These could comprise, for example, experience sampling methods to get insights into users’ decision-making strategies in their daily life^48^. Alternatively, randomized field experiments could be used to compare SAAs with other self-triage decision support tools like telephone triage hotlines^49^.

Alongside the performance metrics and the type of cases used in testing SAAs and LLMs, comparability of evaluation studies requires additional methodological standards for the number of inputters, the gold standard assignment, and the number of self-triage levels that are reported. Although recent studies provide specific recommendations for these issues^20,39,40,50^, they are rarely being applied. Specific recommendations include Meczner et al.’s standards for dealing with inputter variability based on using standardized instructions, multiple inputters, and a pooled accuracy metric to reflect the recommendations that multiple inputters receive^50^. El-Osta et al. standardized the gold standard assignment process concluding that pooling decisions of two independent physician panels gets closer to the best solution than using one physician panel or a single person only^39^. And Kopka et al. reviewed the metrics reported in other studies and proposed a set of standardized metrics to better understand the strengths and weaknesses of an SAA^40,47^. Lastly, standardizing the number of self-triage levels could improve comparability both within and between studies. Most studies use three or four levels, yet not all SAAs provide an “urgent care” recommendation^15^. We suggest that using three triage levels—as originally proposed by Semigran et al.^2^—might increase comparability.

Finally, comparability of evaluation studies requires full transparency regarding the training data and the technologies underlying an SAA or any AI-based triage tools. Our review identified some studies that use established rule-based guidelines^2,7^, but the majority did not include information on how the included tools were developed and which sources the SAAs used. If they mention any database at all, it is described vaguely as using “various medical resources” or “expert input” without providing further details. This lack of transparency raises concerns about the quality of the recommendations and explanations given by these tools. For example, a recent report revealed that the SAA Symptoma used information from a non-evidence-based website on homeopathy to advise users^44^. Without a clear understanding of how and on what data SAAs are trained, it is difficult to assess their performance and safety beyond cases that they are (independently) tested with. This is particularly problematic for explanations of diseases or recommendations that are typically not assessed as part of SAA evaluations. Thus, we think that the field would benefit from more transparency about the sources developers use when creating SAAs.

Once implemented, standardized evaluation and reporting guidelines may be helpful in developing a high-quality certification process. Although some SAAs already comply with medical device regulations in the US and the EU, there is thus far no certification process for SAAs and self-triage tools^31^. Given the high variability between different tools, a standardized certification process by regulatory bodies could help healthcare systems implement only well-performing SAA and can help users avoid low-performing SAAs. The European Union (EU) AI Act promises to lay the groundwork for such a certification by regulating AI-enabled tools such as SAAs and LLMs, for example, by providing a framework to train and test tools under real-world conditions^51^. However, a framework that enables testing is of no use without a clear procedure for high-quality SAA evaluations. As most studies differ significantly in their methodology and often use case vignettes without including real patients, they are insufficient for generating high-quality evidence regarding the effectiveness and safety of SAAs and other automated self-triage and diagnostic tools. To develop evaluation standards, several projects are underway in the EU and globally, such as the CORE MD (Coordinating Research and Evidence for Medical Devices) project^52^, the EDiHTA (European Digital Health Technology Assessment) Framework, the ASSESS-DHT project^53^, or the Global Initiative on AI for Health by three United Nations agencies^54^. These initiatives promise to deliver standardized procedures for future high-quality evaluations for SAAs, LLMs and other digital triage tools that could ultimately result in a certification process.

The conclusions drawn based on the above review have several limitations. First, unlike previous systematic reviews, we focused solely on self-triage accuracy rather than diagnostic accuracy. This choice was motivated by the relevance of a self-triage use case for laypeople. Whereas a preliminary diagnosis might lead to further information-seeking, a final diagnosis often requires medical tests or more details that are not accessible to laypeople^55^. Ultimately, diagnoses are made by medical professionals. As noted in several studies already, aiding laypeople in finding the most suitable care pathway is a more effective use case for these tools^2,18^. This perspective is also reflected in the included studies. Only one study involved laypeople assessing their diagnostic accuracy^30^—unlike numerous studies on SAAs that typically evaluate both diagnostic and self-triage accuracy^18,19^.

Another limitation concerns the unequal number of studies included for SAA, LLM, and laypeople, respectively. Although many studies test the accuracy of SAAs, only few studies examine the accuracy of laypeople let alone the combined accuracy of laypeople using SAA. A potential reason might be the novelty of the field, and that researchers initially focus on evaluating the technological aspects before progressing to more realistic scenarios that include human participants. Similarly, the number of studies evaluating LLMs was also low. Because LLMs were first released with ChatGPT in 2022, the technology can be considered relatively new and there has been limited time to conduct and publish studies on their accuracy. Although there is a vast body of medical research on LLMs, most of it has focused on their ability to pass pre-specified exams like board tests or other diagnostic tasks^23,56^. As more time passes, we can expect to see additional evidence on the self-triage accuracy of LLMs. This is particularly relevant because LLMs seem to quickly improve their accuracy across various tasks with new iterations^57^.

Next, the methodologies varied among the included studies, which complicates direct comparisons of accuracy estimates. Although differences in methods are likely more pronounced for diagnostic accuracy—e.g., some studies evaluate only the first diagnostic choice while others consider the top 3, 5, or 10 choices^18,19^—a wide variety of methods is also used to evaluate self-triage accuracy. A major issue concerns the use of fictitious vignettes that were phrased by clinicians and developed based on clear-cut case descriptions derived from textbooks or physicians’ experiences. Because these vignettes do not accurately reflect real cases that SAAs are approached with^20,32,58,59^, generalizability of most included studies is questionable.

A final limitation refers to the context of use that has been investigated. Based on the studies we identified we can conclude what would happen if SAAs and LLMs led to self-triage decisions without interference of the user, and how laypeople would make self-triage decisions unassisted. In real life, however, it can be assumed that laypeople use various support tools individually or in combination including SAAs, LLMs, the internet (i.e., search engines or social media), telephone triage hotlines, or ask other people for advice^41,60–62^. Thus, the accuracy estimates described in the literature do not reliably reflect self-triage performance in the real world. In the future, differentiating between SAAs and LLMs might become even more complicated. As SAA developers frequently claim to use AI—often without a clear explanation on how it is being used—there may be some overlap with LLMs. Many healthcare technology developers start integrating LLMs into various tools^63,64^, and future SAAs may function similarly and serve as an interface for accessing LLMs. Conversely, some LLM applications may receive a knowledge base and rule-based algorithms (as SAAs currently do) to analyze symptoms in a more transparent way. Thus, although differentiating performance estimates between SAAs, LLMs and laypeople is currently feasible, these two technologies can be expected to merge to some degree in how they will be used in the future.

In conclusion, the triage performance of SAAs varies compared to laypeople’s self-triage decisions; some SAAs outperform laypeople, while others do not. Thus, although SAAs cannot be universally recommended, well-performing SAAs warrant further investigation and should be recommended for use once their safety is assured. LLMs showed less variability and higher accuracy than many SAAs in handling both emergency and non-emergency cases, which suggests a potential usefulness in these scenarios. However, they rarely recommend self-care and cannot be universally endorsed either.

Deciding which tools to use to support laypeople’s self-triage decisions should be based on the specific use case. For users confident that their symptoms require medical attention, a high-performing SAA or LLM may be beneficial. However, for those uncertain whether their symptoms warrant medical attention, most SAAs do not yet effectively differentiate between self-care and medical care, and current LLMs do not provide any assistance in this decision-making process. Although SAAs and LLMs cannot be generally recommended, their use should not be outright discouraged. Many SAAs performed better than the average layperson in multiple studies, and they may have the potential to improve laypeople’s decisions and lead them to more safe decisions. However, before making a final judgment on the utility of SAAs and LLMs, their safety should be assessed in more detail. To do so, future studies should adhere to a standardized evaluation and certification process to identify SAAs and LLMs from which users can benefit. Identifying such tools should always consider the specific use case that is being evaluated, be it for emergencies or for helping patients identify and treat low-acuity symptoms at home.

## Methods

### Eligibility criteria

This study was preregistered on PROSPERO (ID: CRD42024563111) and adheres to the PRISMA reporting guideline^65^. Following a previous systematic review on SAAs^19^, we included studies published from 2010 onward. We included all primary research articles (including preprints) that were published in English. Our inclusion criteria comprised all patient demographics (including both vignette-based studies and real-world evidence studies) and various symptoms, but we excluded studies that focused solely on highly specialized tools or cases, such as SAAs focusing on COVID-19 cases only^66^. Article inclusion required an intervention that examines the self-triage advice of SAAs, LLMs, or laypeople. We excluded any studies that evaluated multiple tools being used simultaneously (e.g., SAAs combined with a telephone triage hotline) or tools that did not offer self-triage advice. To be able to evaluate and compare accuracy, each study needed to include a gold standard solution (i.e., a medically correct triage decision) for the test cases it included. Studies that only rated the appropriateness of the received self-triage advice (e.g., on a 5-point Likert scale) without providing a correct solution were excluded. Lastly, studies were required to quantitatively report (self-)triage accuracy by advising the most appropriate care facility^2^. We excluded any studies that exclusively reported triage accuracy for emergency departments (e.g., using the Manchester Triage Scale or Emergency Severity Index) without considering other care facilities. Studies that reported only diagnostic accuracy without corresponding self-triage accuracy were excluded as well. For the synthesis, we grouped studies according to the agent for which they provided self-triage accuracy estimates, i.e., for SAAs, LLMs and/or laypeople.

### Search strategy and information sources

We conducted our search on July 09, 2024, using the databases Web of Science, MEDLINE/Pubmed, and Scopus to identify relevant articles. The search was limited to studies published from 2010 onward and included English articles only. We developed an initial search string based on previously published systematic reviews of SAAs^17–19^ and adapted it to focus on self-triage accuracy and to include LLMs and laypeople. This search string was refined until it identified all studies reporting self-triage that previous systematic reviews identified. The same refined search string was applied across all databases. All search strings used can be found in the Supplementary Note 1. As an example, the search string for Web of Science read:

AB = (app OR apps OR application OR artificial intelligence OR AI OR online OR web-based OR chatbot OR mobile OR computer-assisted OR internet OR smartphone OR phone OR web) OR AB = (symptom checker OR symptom check* OR symptom assessment app* OR symptom-assessment app* OR webmd OR symptomate OR ada OR yourmd OR mediktor OR buoy OR self-refer*) OR AB =(human OR layperson OR laypeople OR lay OR user OR non-professional OR non-clinician) OR AB = (GPT-3 OR ChatGPT OR GPT-4 OR GPT-4o OR Large Language Model OR LLM OR Claude OR Google Bard OR Mistral OR GPT)) AND AB =(self-triage OR triage OR symptom urgency OR dispositional advice OR self-assess*) AND AB = (accuracy OR correct)

After identifying relevant articles, we conducted both forward and backward citation searches to identify additional studies, particularly preprints, that were not initially retrieved from the databases.

### Data extraction and data analysis

The studies were retrieved and imported into PicoPortal, where they were deduplicated. The titles and abstracts were screened by two researchers (MK & NvK) independently on PicoPortal. In cases of disagreement, both researchers re-examined the title and abstract and resolved conflicts through discussion. Afterwards, the full texts of each eligible study were independently screened by both researchers according to the pre-specified inclusion and exclusion criteria. Cases of disagreement were examined again, and conflicts were resolved through further discussion.

The data were extracted by both researchers independently using a standardized Excel template. The primary outcome focused on assessing the self-triage accuracy of SAAs, LLMs, and laypeople. For the secondary outcomes, the researchers extracted PICOS (Population, Intervention, Comparison, Outcome, Study Design) variables^67,68^, the reported accuracy across the urgency levels, and the specific self-triage accuracy of each SAA, LLM, or layperson population. To gain insights into differences in methodology, the data extraction form included the number of SAAs, LLMs, and laypeople, a brief description of the methods used, the number of triage levels in the study, the number of cases examined, the gold standard assignment process, the number of data inputters, other reported outcomes with respect to self-triage, the algorithm and data basis of the SAAs included, as well as any conflicts of interest and funding sources. Any instances of missing data were coded as “*not available”*. Due to the varying methodologies among the included studies, the data were analyzed using narrative synthesis, as the estimates of the studies are not directly comparable. Nonetheless, a quantitative summary of the accuracy across all included studies is provided to show overall trends.

### Risk of bias

The risk of bias was assessed by two authors (MK & NvK) independently using the Quality Assessment of Diagnostic Accuracy Studies-2 tool (QUADAS-2)^69^. This tool helps researchers conduct systematic reviews on diagnostic applications and procedures to determine the risk of bias and applicability of included studies. It uses four dimensions to rate the risk of bias and three dimensions to rate the applicability of a study to the research question. The risk of bias and applicability concerns were categorized into “low”, “high”, and “unclear”. Any discrepancies between the two raters were again resolved through discussion.

## Supplementary information

Supplementary Information

## Data Availability

The data was analyzed using R 4.3.3 and the tidyverse packages v2.0.0. The code for data visualization is available upon request.

## Data availability

No datasets were generated or analysed during the current study.
